# A View of the Formation, Discipline, and Economy of Armies

**Published:** 1846-01

**Authors:** 


### ART. XII.

*A View of the Formation, Discipline, and Economy of Armies.* By the late ROBERT JACKSON, M.D., Inspector-General of Army Hospitals. *The third edition, revised, with a Memoir of his Life and Services, drawn up from his own papers and the communications of his survivors. London,* 1845. 8vo, pp. 560.

THE volume before us consists of two parts, the first being a Memoir of Dr. Jackson; and the second, a new edition, his work on the Formation, Discipline, and Economy of Armies. We shall notice them in order.

ROBERT JACKSON was one of a large class of men of whom Scotland has just reason to be proud, and who themselves have yet greater reason to be proud of Scotland—men who, by the strength of their native intellect and the goodness of their moral qualities, have, under the fostering care of their country's institutions, raised themselves from poverty and low estate to a high, dignified, and influential position in society, where they were enabled to do infinitely more good to their fellow-men and their native land, and to enjoy more personal happiness than could possibly have been their fortune in the station in which they were born. It is only in this increased capacity of doing good to others, as well as of augmenting the stock of individual happiness, that we see any benefit in the raising or being raised from a low to an elevated station; as we cannot regard anything as intrinsically and absolutely superior or inferior in either one or the other, or in the occupiers thereof—feeling and acknowledging with the mightiest of Scotland's low-born great, that

> "The rank is but the guinea's stamp,
> The man's the gowd for a' that."

And it is only in the departments of literature and science, or in the professions based upon these, that the full worth of the Scottish character and the peculiar excellence of Scottish institutions can be truly manifested. In commerce and the arts, ordinary industry and prudence, together with a favorable concurrence of circumstances, may and do raise men in all countries from humble poverty to wealth and distinction, with very little aid from letters, and without any very extraordinary personal exertions. And this takes place nearly as often in England as in Scotland. But with a few very remarkable exceptions, we shall look in vain in the learned professions for Englishmen who have reached the highest posts and honours from the very lowest stations of society. The case is, however, very different with Scotsmen; as we believe it will be found that many of the most distinguished and estimable members of the learned professions, more especially of the medical profession, have sprung from the humblest grades of life, from the low but not unenlightened abodes of the small farmer, the artisan, or even the cottar. And the causes of the difference are sufficiently obvious. They lie, partly, in difference of national character—particularly in that noble ambition of every Scottish father to raise his son above himself—but chiefly in the difference of the educational establishments of the two countries. The existence in every parish, or at least in every small district, of a good school for teaching not merely the rudiments of ordinary knowledge but *the classics,* and at a rate of expense consistent with the resources of the industrious in every rank of life, keeps up, throughout the length and breadth of SCOTLAND, as it were, perpetual lamps of wisdom, which serve, at once, as tests for distinguishing the capacities of all her youth, and as sources of illumination to the minds of the select few who are found worthy to tread in the higher paths of knowledge. How different the case is in England need not be here stated; nor is this the place to inquire what is the relative value of the respective systems of the two countries: but we may surely be allowed to regard the failure of well-meant but most inadequate measures to introduce a national system of instruction into England, as a proof that the importance of education is altogether unknown or misunderstood by those who have themselves most profited by education in that country.

I. Dr. Jackson was the son of a small Scotch farmer, and was born at Stone Byers, Lanarkshire, in 1750. He was educated at one of the parish schools above referred to. After leaving school he served a three-years' apprenticeship to a surgeon at Biggar, and in 1768 went to the university of Edinburgh to study medicine, where he attended the classes during three winter sessions; making a voyage to Greenland each summer on board a whaler, as the medical officer, with the laudable purpose of thereby assisting to provide the means for pursuing his studies. Having gone out as a passenger to Jamaica, in 1774, he became assistant to Dr. King at Savanna-la-Mar, where he first was introduced to military practice, having medical charge of a portion of H. M.'s 60th regiment. In this situation he remained about four years until, actuated by a love of change and enterprise, which seems to have adhered to him throughout life, he resolved to proceed to America to push his fortune there. "It could not be said that he was happy at Jamaica: the scene was much too limited for one of his adventurous and inquiring turn of mind." He accordingly embarked for New York, but having neglected to provide himself with a certificate of having paid all his debts, which was required by the laws of the island, he was landed at Point Morant, the eastern extremity of Jamaica. His finances not admitting of his hiring a horse, he was compelled to make his way on foot from Kingston to Savanna-la-Mar, 130 miles distant; an undertaking, in such a climate, of a very arduous nature. Having accomplished this journey, and provided himself with the necessary document, he again embarked for New York, where he arrived in good health, but with an empty purse, in 1778. Failing to procure a situation as mate at the general or naval hospitals there, he offered his services as a volunteer to the 71st Highlanders, encamped about seven miles from New York, which were accepted; and as he stated he had studied medicine, he was appointed to act as surgeon's mate, at least until a commission should fall vacant.

He continued to serve as hospital-mate with this regiment till he was taken prisoner at Cowpens, in January, 1781, under circumstances most creditable to himself. He was released on parole early in 1782, and returned by New York to England, and resided in London till peace was concluded between Great Britain and America in 1783. He started in May of that year on a pedestrian tour through France, Switzerland, Italy, and Germany, which occupied seven months. On the 9th December, 1783, he was gazetted to an ensignсy in the 71st regiment; and in the January following he walked from London to Perth to join his old corps, which was then about to be disbanded. He must have been almost immediately afterwards placed on half-pay by reduction, which he continued to enjoy till promoted to be surgeon of the 'Buffs' in 1793. In 1784 he married, at Edinburgh, the daughter of Dr. Stevenson, "an accomplished woman of good fortune. Being by this union placed in easy circumstances, he was enabled to pursue his studies at Paris for a longer period than on the former occasion." In the end of the same year he went to Brussels, and thence to Leyden, where, in 1785, he took the degree of M.D., and shortly afterwards established himself in practice as a physician at Stockton-upon-Tees, Durham.

In 1793 he offered to serve again in the army as physician to the forces, but as he had never served as surgeon, his offer was not accepted. He was, however, appointed to the surgeoncy of the 3d regiment or 'Buffs,' with a promise from Mr. Hunter, who was then at the head of the department, that the physician's commission would be given him on the first proper opportunity. In 1794 he accompanied the regiment to Flanders, and in the end of that year received his promotion by order of the Duke of York, notwithstanding the opposition of Sir Lucas Pepys, the physician-general, who declined to carry out the arrangement of Mr. Hunter. Shortly afterwards he succeeded Dr. Kennedy as inspector of hospitals, and was thus placed in charge of the medical department of a division of the army. On the infantry being withdrawn from Bremen, he was ordered to England. In 1796 he accompanied the expedition to St. Domingo as second medical officer, Mr. Weir being the first, and served there till the evacuation of Port-au-Prince by the British troops in 1798. He thence embarked for America, and made a tour through the States with Dr. Borland, returning in the same year to England, and rejoining his family at Stockton. In 1799, at the desire of the Russian ambassador, he was placed in charge of a body of Russian troops stationed in Guernsey and Jersey, (who, after their service in North Holland, had arrived in a most sickly condition,) and obtained much credit for the able manner in which he conducted their hospitals.

In 1800, His Royal Highness the commander-in-chief nominated him to be "physician and head of the army-depôt hospital," (at the Isle of Wight.) In the latter end of 1801, the physician-general and surgeon-general preferred charges of malpractice against Dr. Jackson, which, on being investigated by a board, were declared to be groundless, and they received a merited reprimand. As Dr. Jackson did not not choose to hold intercourse with a person who had acted as he conceived Mr. Keate to have done, and as he must have corresponded with him had he remained at the depôt hospital, he tendered his resignation, which was accepted by the commander-in-chief, who, in his letter noticed in a very flattering manner the doctor's services. Shortly after this he published two works on the medical department of armies, which assisted to draw attention to the subject, and ultimately to bring about the abolition of the board as it then existed, and the adoption of the present system. These works were entitled 'Remarks on the Constitution of the Medical Department of the British Army, with a detail of Hospital Management,' (8vo edition, 1803,) and 'A System of Arrangement and Discipline for the Medical Department of Armies,' (8vo edition, 1805.) In 1807 he was offered and accepted the situation of military secretary to General Simcoe, the commander-in-chief in India,—"a rare, if not a solitary instance of the selection in the Royal Army of a medical officer to such a situation." Unfortunately, General Simcoe did not live to proceed to his command.

The physician and surgeon-general of the army repeated, in 1809, their accusations of 1801, which had already been declared unfounded; but, on Dr. Jackson applying for redress by a court-martial, he was informed it could not be granted as he was on half-pay. In the excitement of the moment he took the law into his own hands, and caned Mr. Keate, the surgeon-general, for which he received six months' imprisonment in the Marshalsea, and was required at the expiration of that period to give security for his good behaviour for three years.

The medical board having been dissolved in 1810, Mr. Weir was appointed Director-General. Having had experience of Dr. Jackson's services in St. Domingo, he applied for and obtained leave to employ him as inspector of hospitals in the West Indies, where he served with credit till 1815. From this period he remained unemployed till the autumn of 1819, when he offered to proceed to Cadiz to investigate the question of the contagious or non-contagious nature of the epidemic fever prevailing in that place. Being prevented by political disturbances from reaching Cadiz, he proceeded—actuated very much by that love of change of scene which, as we have seen, characterized his younger days—to Constantinople, Smyrna, the Archipelago, and Athens, returning by Patras and Zante to Malta and Gibraltar. Hence he proceeded to Cadiz, where the epidemic was raging, and remained two months studying its characters. He afterwards went to Xeres, where the disease was more severe and more fatal, and on his return to England published the result of his observations. This was the last public service on which he was ever employed, although in 1827, being then seventy-six years old, he offered to proceed to Portugal with the British force sent out under Sir William Clinton. A few weeks after this he died of paralysis at Thursby, near Carlisle.

Such is a brief outline of the principal events in the life of one to whom the army owes much for the improvements he effected in the military hospitals, as regards the comfort and welfare of the soldier, and who, at the same time, introduced a system of economy into their management, which has saved large sums of money to the country. His biographer states that "his plan, substituted for the colonial contract alone, saved £80,000 to the British government."

One of the most prominent features in Dr. Jackson's character, was his untiring perseverance in endeavouring to improve the condition of the soldier, to preserve him in a state of health and efficicncy, and to afford him the best assistance in the hour of sickness, notwithstanding the powerful opposition of interested parties. He was remarkable also for the practical nature of the improvements he proposed in the department, and for the disinterested manner in which he was willing to sacrifice personal considerations for the good of the service. On more than one occasion he intimated his willingness to serve wherever he could be useful, "*and that it was a matter of indifference to him in what rank he served;*" and when he volunteered to accompany the expedition to Portugal in 1827, he offered to waive his rank, and do duty in the military hospitals under a junior.

Jackson appears to have agreed in opinion with Paracelsus, when he says "art does not go after any one; it is we who must follow her. I have somewhere heard that a physician must be a rambler, and it is just that which I like best. Diseases travel everywhere over the wide world, and the doctor who would know much must travel much. Whoever will indagate nature must trample her book with his feet." We cannot, however, agree with his biographer, that "the details of Dr. Jackson's wanderings on the continent may serve to encourage those who cannot purchase instruction at the great seminaries of learning, to seek it where they can, in the wide field of nature, where it costs little but the exertion of seeking, and it has the recommendation of being always genuine." We hold the advantages Dr. Jackson derived from his rambles to be chiefly attributable to great innate capacity, and a remarkable power of observation, aided probably to some extent by his previous education and habits. It would be unsafe to draw any such conclusion, therefore, from this instance;—it would, in fact, be arguing from the exception instead of the general rule.

We could have wished more care bestowed on the literary execution of this Memoir, which in some parts reminded us of the remark applied by King James, of facetious memory, to Lord Bacon's works, that "like the peace of God, they pass all understanding." A little more labour in the compilation would also have materially improved the work; there is a great want of dates, and when given they are not always correct. For instance, in narrating his tour in the beginning of 1783, the editor observes, "his pecuniary means for the undertaking were but low. As a hospital mate, he was entitled to no half-pay; but he had been for years an ensign in the 71st regiment," &c. Now he was not gazetted to an ensigncy in the 71st till 9th December of that year, after he had returned from the continent; an anachronism which ought to have been avoided.

The most serious fault, however, we have to find with the Memoir, and which detracts greatly from its value, is the omission of a review of the author's works. The editor states, (p. 116,) "This is not the place to speak of his professional publications, which we briefly dismiss by saying that they are characterized by vigour of thought, close observation of nature, and originality of views." Now, from this opinion we dissent, because we know of no place so suitable for a review of the opinions and publications of a literary or professional man, as a memoir of the kind now before us, especially when it is not compiled till a sufficient period has elapsed after his death to deaden those deep regrets we feel at the loss of one we respect or love, and thus to enable us calmly to exercise our judgment in the performance of the task. To the Memoir is appended a list of his works, from which, however, two have been omitted, viz. 'A Sketch (*analytical*) of the History and Cure of Contagious Fever,' (London, 8vo, 1819;) and 'Remarks on the Epidemic Yellow Fever, which has appeared at intervals on the south coast of Spain, since the year 1800,' (London, 8vo, 1821.)

II. The work on the 'Formation, Discipline, and Economy of Armies,' was published at Stockton in 1804. Dr. Jackson states the main object to be "to investigate the powers and qualities of the physical properties of man individually, according to classes and nations; to impress the utility, and to suggest the means of arranging the parts in the military fabric, according to the powers of exertion rather than the semblance of external figure; to animate the fabric so formed, by the strongest and most extensive principle which operates upon animal structure, and to preserve the fabric so arranged and animated, in a state of efficiency, by the observance of a correct system of economy." His hobby was, in short, to make efficient soldiers without much reference to human passions, or to the individual happiness of the men. Indeed, he sometimes seems to convey the idea that he thought men were created for the purpose of being made soldiers—intellectual instruments—attending to their physical wants for the purpose of sustaining and improving their physical powers, on the same principle that care is bestowed on cavalry horses; and regarding the cultivation of their intellectual or moral qualities in relation only to their military efficiency, and not as promoting their welfare, and ultimate happiness, as individuals. A second edition was brought out in 1824, in the preface to which he alludes to the probability of a charge of presumption being brought against the physician who ventures to give instruction on the mode of forming, training, and maintaining the instrument of war in discipline and efficiency. But he most justly remarks that "an estimate of materials is primary to the exertion of the military, as well as other fabric; and as medical men are, or ought to be, from the nature of their studies, better acquainted with the materials of which armies are composed, than men of other professions, the author is not disposed to admit the charge of encroachment,—not even to allow that he has exceeded the limit of his station in doing what he has done."

The work is divided into six parts. The first treats of the "estimate of qualities, and selection of military recruits," which he considers as affected by class or race, climate, locality, age, state of society, pursuits and occupations; and lastly, the powers and capacities as existing in the actual constitution of the individual. In this part he discusses the various qualities which peculiarly fit men for different arms of the service, and offers many excellent remarks on the classing of men according to their previous occupation and habits, and their physical powers, as ascertained by trial, and thus endeavouring to obtain correspondence of action rather than uniformity of appearance. Unfortunately, however, these views cannot be reduced to practice in the British army, recruited as it is by voluntary enlistment. In this respect, commanding officers are in much the same position as the army surgeon is in the selection of recruits, regarding which Dr. Jackson justly observes, " the duty imposed in this case is not to select what is in every way good, but to reject what is absolutely unfit." The second part consists of a sketch of the military character of the nations which are, or have been, most eminent in war. Part third gives an outline of tactic, or rudiment of military training. Part fourth treats of the intellectual and moral motives of military action. These contain much interesting and instructive matter, but as they relate more to military than medical subjects, we pass on to part fifth, on " discipline and economy for the field and quarters," under which are considered the important subjects of diet, clothing, exercise, barracks, camps, and transport-ships." On these points the health of our troops materially depends, and they consequently deserve the attention both of the military and medical officer. " The medical history of armies," says our author, " holds out a dismal picture of human misery. Armies were crippled, almost annihilated, by artificial diseases in the late war, that is, by contagious fevers, proceeding from corrupted sources of recruiting, and gaining strength from ignorance of the principles which conduce to the preservation of health, or from indifference and negligence in applying them to the occasion. Such losses are melancholy, because they proceed from error. The errors are not always reprehensible, for they proceed from ignorance and misapplied care, as often perhaps as from indifference and neglect. Soldiers are selected from the healthy part of the community. Reason says they ought to be more healthy than the mass of the people; it is not so in fact. The cause of sickness does not consist in actual hardship, for that is rarely to a great extent, or where it does occur it rarely affects the health." Dr. Hunter has justly remarked, that " in military physick, the great improvements to be made are not so much in the cure as in the prevention of diseases." (Diseases of the Army in Jamaica in 1766.)

Dr. Jackson's remarks on the various topics treated in this Part are in general very judicious, and evince a thorough knowledge of his subject—a knowledge acquired under the diversified circumstances of service in the field, and in garrison, at home, and abroad, in tropical and in temperate colonies. On one or two points we differ in opinion from him, but his observations are all well calculated to suggest useful hints, and afford materials for reflection. Under the head of diet he discusses a point worthy of the consideration of the military authorities, recommending that " care should be taken, in laying the basis of primary education, that every one be competent to dress in a suitable manner the raw provisions of which the ration consists. If the principle according to which the application of heat acts on raw provisions, for the improvement of their taste and nutritive qualities, be explained to the young soldier, and rightly comprehended by him, he will know to vary the mode according to his means, and not complain that his meal is unsavory when his ration is good in kind and abundant in quantity; a case which often happens with those who are young and uneducated, and which happens with almost all British soldiers in their first campaign." It is well known that in the last war the French troops much excelled the British in this respect. On service this is not a matter of such small account as some may suppose, for by enabling the men to have their food in a palatable form they are induced to eat their rations, when from great fatigue they might otherwise reject it if in an uninviting shape. "It may be said without offence that if the art of cookery be understood by the English nation, it is not generally practised by the English soldiery."

Our space will not permit us to review in detail the opinions of our author on the various circumstances likely to affect the health of soldiers, and the best means of maintaining them in a state of efficiency—nor is it necessary. The work before us deserves the careful perusal of every medical officer in the army, and might be advantageously studied by every commanding officer. As the success of any military operation must depend in a great measure on the health and vigour of the troops employed to carry it into execution, there can be no subject more deserving the attention of officers than the best means of warding off disease from them, and of keeping them healthy and efficient.

We have already observed that Dr. Jackson was remarkable for the practical character of his suggestions, and for his zealous endeavours to promote the comfort and welfare of the soldier. We cannot illustrate this better, or point out more forcibly the great credit due to him, than by enumerating briefly some of the suggestions he made in the first edition of his work in 1804, and showing how these have been gradually adopted in the army. As regards diet, he recommended the introduction of a breakfast mess, which is now the rule of the service; he wrote strongly against the issue of a spirit ration as destructive of health and morals, and injurious to discipline: this was finally abolished by Sir H. Hardinge in 1830. In dress and equipment, he recommended the reduction of the weight of the kit, which has been partially effected, but is still capable of improvement; the use of trousers instead of breeches and leggins; the abolition of the custom of dressing the hair with grease and flour, a custom now unknown, and of the use of pipeclay to clean the clothing. On one point connected with soldiers' clothing, there yet remains great room for improvement. "The common manufacturer," says Jackson, "exerts his genius to produce a commodity of the flimsiest interior texture with the best exterior surface. The contractor for soldiers' clothing exerts himself to go beyond the manufacturer for the common market, and he generally succeeds; for soldiers' clothing is inspected and approved by less competent judges than those who purchase for themselves. It thus happens, from the bad quality of contract clothing and contract shoes, English soldiers are sometimes ill clothed on actual service, and almost always ill shod . . . . a circumstance which, exclusive of better primary education for war, gives foreign soldiers great advantages over English, in protracted campaigns and harassing services carried on at a distance from the depôt of stores."

In discussing the influence of exercise on the health, he advises the encouragement of dancing, running, football, cricket, quoits, and those other manly and athletic games, the introduction of which into the army has since been strongly recommended by the Military Punishment Commission, and partially carried into effect. But while he thus desired to find employment for the body, he also inculcated the necessity of attending to the mental culture of the soldier, a subject which has been slowly but steadily making progress, and now receives the sanction of the highest military authorities.

We shall only notice one more suggestion, that of quartering the troops in the West Indies in the mountains, and employing the soldiers to erect hut barracks for themselves in these situations. This he strongly advocated on the plea of humanity, economy, and increased military efficiency. Upwards of a third of a century afterwards we find these views adopted by Government, and carried out in Jamaica by the very means he suggested, not however until the policy of the measure had been demonstrated by statistical investigation, and enforced by the loss of upwards of 12,000 soldiers, of whom it is probable two thirds *at least* might have been saved had the measure been adopted when he first proposed it. Although he did not live to see this or the abolition of the spirit ration, we can conceive the satisfaction he must have experienced at the steady progress the principles he had laid down were gradually making in the army, —a satisfaction in a great measure arising from the conviction that they were promoting that which had ever been the chief object of his exertions, the melioration of the condition of the soldier. We cannot conclude this article better than with the following quotation from the preface to the second edition:

"The subject of the present work has been under the author's consideration for upwards of twenty years. He has looked at it without prepossession, as desirous to ascertain the truth. He believes that many of the hints which have occurred to him would tend, if properly understood, to diminish the miseries which are common in military life; and in that belief he has put them together, and now presents them to the public, gratified if they do good; at any rate satisfied with himself, as acquitted of a duty which he conceives to belong to the station in which he has acted."

---

